# Protruding hydrogen atoms as markers for the molecular orientation of a metallocene

**DOI:** 10.3762/bjnano.11.127

**Published:** 2020-09-22

**Authors:** Linda Laflör, Michael Reichling, Philipp Rahe

**Affiliations:** 1 Fachbereich Physik, Universität Osnabrück, Barbarastrasse 7, 49076 Osnabrück, Germany

**Keywords:** calcium fluoride (CaF_2_), ferrocene, functionalised tips, high-resolution imaging, non-contact atomic force microscopy

## Abstract

A distinct dumbbell shape is observed as the dominant contrast feature in the experimental data when imaging 1,1’-ferrocene dicarboxylic acid (FDCA) molecules on bulk and thin film CaF_2_(111) surfaces with non-contact atomic force microscopy (NC-AFM). We use NC-AFM image calculations with the probe particle model to interpret this distinct shape by repulsive interactions between the NC-AFM tip and the top hydrogen atoms of the cyclopentadienyl (Cp) rings. Simulated NC-AFM images show an excellent agreement with experimental constant-height NC-AFM data of FDCA molecules at several tip–sample distances. By measuring this distinct dumbbell shape together with the molecular orientation, a strategy is proposed to determine the conformation of the ferrocene moiety, herein on CaF_2_(111) surfaces, by using the protruding hydrogen atoms as markers.

## Introduction

It is still a challenge to determine the precise adsorption geometry of three-dimensional (3D) molecules on surfaces. While spectroscopic methods, such as X-ray standing waves [1] or photoelectron diffraction [2], can precisely determine the location of atomic species in ordered molecular systems on surfaces [3], scanning probe methods are commonly employed for the investigation of both ordered and unordered molecular systems as well as of individual and isolated species [4–6]. For example, two different non-planar isomers of dibenzo[*a*,*h*]thianthrene molecules could be identified by high-resolution non-contact atomic force microscopy (NC-AFM) [7–8]. Furthermore, the imaging of hydrogen atoms attached to propellane molecules with CO-functionalised tips was suggested to enable the identification of three-dimensional molecules [9]. Very recently, a combination of NC-AFM and automated structure detection has been able to resolve the orientation of small organic 3D molecules from NC-AFM data [10]. Adapting these approaches to a broad range of 3D molecules bears the strong potential not only to gain insight into the on-surface adsorption properties of molecules, but also to unravel the intramolecular geometry.

A prototypical class of molecules with a hindered rotation determines different conformers is the metallocene family. These molecules consist of a central metal atom sandwiched between two rotatable cyclopentadienyl (C_5_H_5_, Cp) rings. The limiting cases of the ring orientations are known as eclipsed and staggered conformers. For ferrocene, the rotational barrier between these conformers amounts to only 0.04 eV in the gas phase [11–13], while for substituted ferrocenes the energy barrier values determined in the gas phase amount up to 0.2 eV [14–16]. In solution, barrier values of up to 0.7 eV are measured [17–18] and for ionised derivates calculations yielded rotational barrier values of up to 1.4 eV [19].

**Figure 1:**
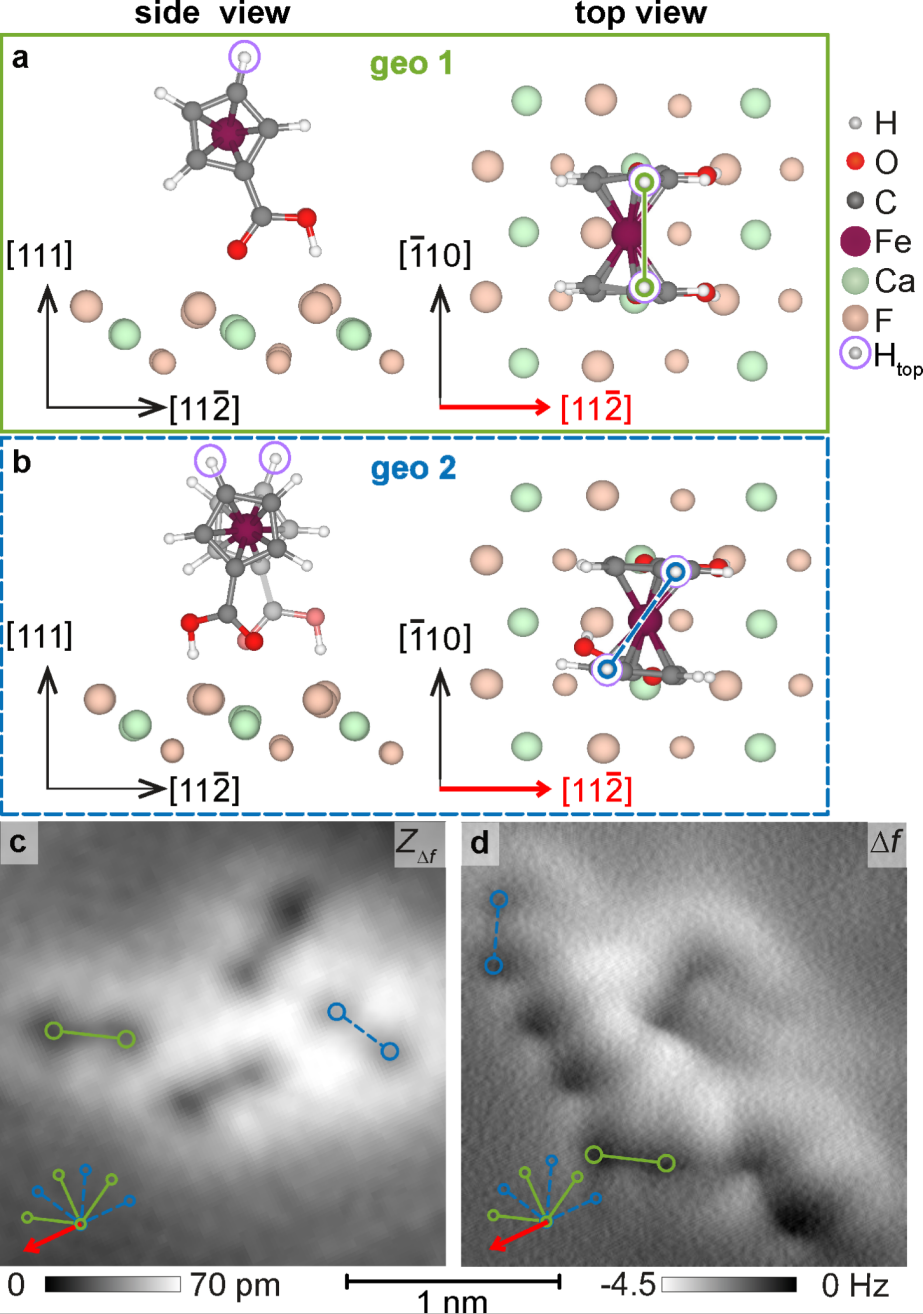
Dumbbell shape of single FDCA molecules. Quadruped binding motif of FDCA on CaF_2_(111) (adapted from [22]) of (a) geo 1 and (b) geo 2. Dumbbell shape of FDCA molecules (c) on bulk CaF_2_(111) measured by NC-AFM in topography mode and (d) on thin film CaF_2_(111) surface measured in the constant-height mode (adapted from [22]). The orientations of the two geometries geo 1 and geo 2 are marked by solid green and dashed blue lines, respectively, and are included in all images with the respective orientation. (A five pixel averaging filter was applied to reduce noise within the frequency shift image. An inverted colour scale is used for the constant-height Δ*f* NC-AFM data to match the topography appearance.)

From this class of molecules we investigate the ferrocene derivate 1,1’-ferrocene dicarboxylic acid (FDCA), a ferrocene functionalised with two carboxylic acid moieties. This molecule has been analysed before on Ag(111), Cu(110), and Cu_3_N/Cu(110) surfaces [20] as well as on the insulator surfaces calcite(104) [21] and CaF_2_(111) [22]. An eclipsed ferrocene conformation was found to be predominant on the metallic surfaces [20] and on calcite(104) [21]. On CaF_2_(111) surfaces, density functional theory (DFT) calculations revealed an optimum geometry of the eclipsed conformer in a nearly upright-standing, slightly tilted fashion, as well as a staggered conformer in an energetically less favourable geometry with one rotated carboxylic acid moiety [22]. These geometries, referred to as “geo 1” and “geo 2”, are depicted in Figure 1a and Figure 1b, respectively. The molecule–surface bond, with a calculated binding energy for geo 1 of −1.74 eV, is surprisingly strong [22]. Due to the tilted molecular orientations, both geometries have two hydrogen atoms as the topmost unit protruding the surface. However, the orientation with respect to the CaF_2_(111) surface lattice differs for the two geometries.

In this work we investigate the NC-AFM contrast formation of FDCA molecules on CaF_2_(111) surfaces. A distinct dumbbell shape has been observed in both topography and constant-height imaging modes in low-temperature experiments (5 and 77 K) with qPlus sensors as well as at room temperature using silicon cantilevers [22]. Although the NC-AFM tips were not functionalised, i.e., not specifically terminated with atoms or molecules for imaging, we find a very good agreement between the experimental data and probe particle model (PPM) [23] calculations using CO and Xe tips. Our analysis suggest that contrast formation is governed by repulsion between the tip and the hydrogen atoms serving as markers for the molecular conformation.

## Experimental

Scanning probe microscopy experiments were performed on CaF_2_(111) bulk and CaF_2_/CaF_1_/Si(111) thin film samples under ultrahigh vacuum conditions (*p <* 5 × 10^−10^ mbar) in two separate systems equipped with appropriate facilities for in situ sample preparation.

Bulk CaF_2_ crystals (Korth Kristalle, Altenholz, Germany) were cleaved in vacuum [24] after degassing the crystal and sample holder. To reduce residual charge on the surface [25], the sample was heated at about 330 K for one hour prior to the deposition of the ferrocene molecules and NC-AFM experiments. Thin CaF_2_ films were prepared by deposition of CaF_2_ (purity 99.9%, Alfa Aesar, Kandel, Germany) from an EFM3T e-beam evaporator (Focus GmbH, Huenstetten, Germany) on freshly prepared Si(111)-(7 × 7) surfaces held at about 930 K. Silicon substrates were highly B-doped p-type Si(111) samples (Institute of Electronic Materials Technology, Warsaw, Poland) with the (7 × 7) reconstruction prepared by flash cycles. Further details on the thin film growth and properties can be found in [22,26–28].

Deposition of 1,1’-ferrocene dicarboxylic acid (purity 98%, Alfa Aesar, Kandel, Germany) on samples held at room temperature was accomplished by sublimation from custom-built Knudsen cells heated to about 390 K.

For bulk samples, the 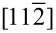 surface direction was determined by cleaving the crystal after the NC-AFM experiments along a (111) plane other than the surface plane. For thin film samples, the orientation of the Si(111)-(7 × 7) unit cell was measured by scanning tunnelling microscopy (STM) and the 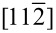 direction was determined by considering the B-type epitaxy of the CaF_2_/CaF_1_/Si(111) thin films samples, see [22,26] for further details.

STM and NC-AFM experiments were conducted at low temperatures (5 and 77 K) in two separate systems. Experiments on bulk crystals were performed using an Omicron LT qPlus gen.III instrument (ScientaOmicron GmbH, Taunusstein, Germany), while experiments on thin films used a ScientaOmicron LT qPlus gen.II machine, both operated with a MATRIX controller. W tips attached to tuning fork sensors in qPlus configuration [29] were used in both systems. For the measurements on thin film samples, custom-built sensors were fabricated with a separate tunnelling current wire to exclude cross-talk between the NC-AFM and tunnelling current signals [30], while sensors as supplied by the manufacturer were used for measurements on bulk CaF_2_ samples. Sensors were excited to oscillation amplitudes between about 0.15 and 0.8 nm. Both instruments were equipped with an atom-tracking system for drift measurement and compensation [31]. All experimental data were analysed using Gwyddion [32].

The probe particle model (PPM) [23] was used for simulating NC-AFM imaging contrast. Neutral oxygen and xenon atoms were used as probe particles and frequency-shift Δ*f* data were calculated for an oscillation amplitude of 0.5 nm. Lateral and vertical stiffness were chosen as 0.5 and 20 N/m, respectively. FDCA molecular models in the DFT-optimised geometries (using geo 1 and geo 2 from [22], see Figure 1a,b) were arranged along the CaF_2_ 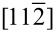 direction, resembling one molecular row of the 
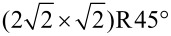
 superstructure that was observed in the experiments. All simulated Δ*f* data are shown with a colour code representing a strong attractive tip–surface interaction as bright and a weak attractive or repulsive interaction as dark to allow for a direct comparison with the experimental data. We refer to this representation as “inverted colour scale”.

## Results and Discussion

Figure 1c and Figure 1d show examples of the prevalently observed dumbbell-shaped appearance of FDCA molecules on CaF_2_(111) surfaces [22] for NC-AFM measurements performed at 5 and 77 K on bulk and thin film samples, respectively. Previously, no difference in the molecular properties when comparing adsorption on CaF_2_(111) bulk and thin film surfaces has been observed [22].

**Figure 2:**
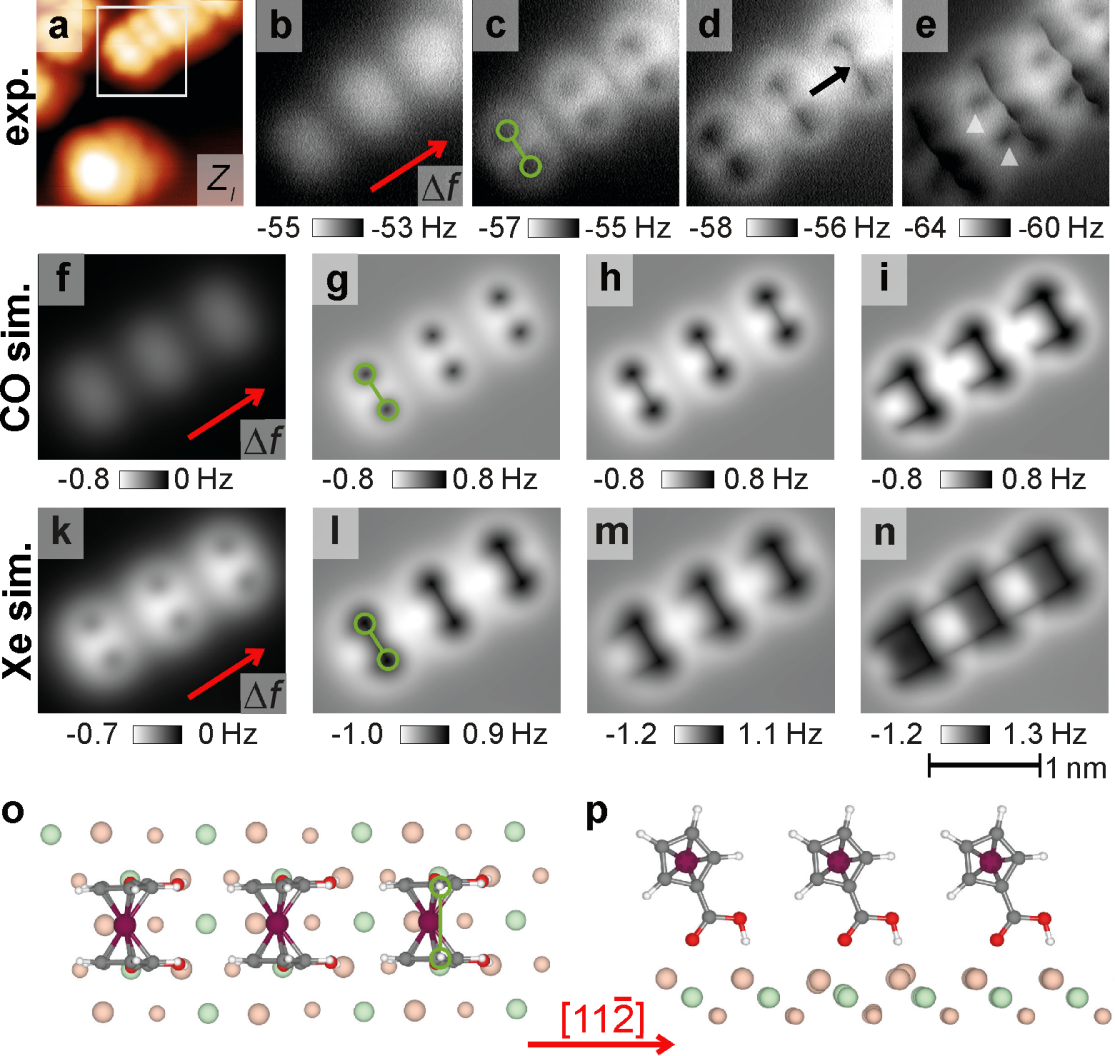
High-resolution NC-AFM imaging and simulation. Experimental and simulated frequency-shift images of a single molecular row along the 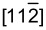 direction (red arrows). Exemplary molecules are marked by a green dumbbell. (a) Experimental STM image (*U* = 4 V, *I_t_* = 2 pA). (b–e) Experimental constant-height NC-AFM data (*f*_0_ = 24363 Hz, *Q*_0_ ≈ 6000, *A*_0_ = 0.27 nm). Frequency shift ranges are shown below each image (inverted colour scales are used). A four pixel averaging filter was applied to reduce noise within the frequency shift images. A slight tilt of the constant-height plane leads to a small modulation along the row. (f–i) Simulated NC-AFM images using a CO tip model. (k–n) Simulated NC-AFM images for a Xe tip model positioned with the tip backbone positioned at the same heights as for the CO image calculations. (o, p) Structure of the simulation cell with each molecule in the single-molecule DFT-optimised geometry geo 1 [22].

The distance-dependency of the imaging contrast was analysed for constant-height data where frequency-shift images were acquired at different tip–sample distances, see Figure 2b–e. Data were acquired above a region where several FDCA molecules were arranged along the 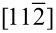 direction (see STM data in Figure 2a), with a molecular separation determined by the CaF_2_(111) lattice periodicity of 669 pm. Upon step-wise reducing the tip–surface distance, the experimental NC-AFM contrast evolves from an elliptic shape caused by attractive tip–sample interactions (Figure 2b) into ring-like structures, followed by the dumbbell shape (Figure 2c). Further reduction of the tip–sample distance then results in the evolution of a sharp line (one example marked by an arrow in Figure 2d), which suggests a relaxation of the front tip apex in agreement with earlier observations in organic [33–34] or inorganic [35–36] systems. For the smallest tip–sample separation, two short elongated dark segments (see markers in Figure 2e) evolve on one side of a sharp line connecting the weights of the dumbbells.

For an interpretation of the NC-AFM contrast, we run simulations based on the probe particle model [23] for three molecules aligned along 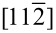, each molecule being in the DFT-optimised adsorption geometry geo 1 [22], as shown in Figure 2o,p. We use Lennard-Jones parameters for CO and Xe tips, a neutral probe particle, and stiffness values of (*k_x_*, *k_y_*, *k_z_*) = (0.5, 0.5, 20) N/m, yielding the results for CO reproduced in Figure 2f–i and for Xe in Figure 2k–n at the same heights. Both series of images exhibit a remarkable similarity to the experimental data. The change from the ellipse to the dumbbell shape is reproduced as well as the evolution of a sharp line connecting the two dumbbell weights (Figure 2h,l). The characteristic contrast features appear in the simulations at different heights for the different tips, a finding which we attribute to the different van der Waals radii of CO and xenon. From a comparison with the molecular geometry we can confidentially assign the dark weights of the dumbbell to repulsive interactions with the top hydrogen atoms H_top_ (see Figure 1a). The bright halo around these two weights is in turn caused by attractive forces acting between the tip and other atoms of the FDCA molecule. Thus, the PPM simulations allow for the assignment of the dumbbell shape to a single FDCA molecule and, in turn, the orientation of the dumbbell precisely defines the axis between the top hydrogen atoms. Additionally, the asymmetry that evolves at small tip–sample distances is reproduced by the simulations, especially the two small, elongated dark segments marked by small triangles in Figure 2e. In the calculations, these dark segments are due to repulsive interactions with the lower-lying hydrogen atoms of the Cp rings, similar to the lines across the top bond of the Cp ring observed in previous measurements of FDCA on Cu(110) and Cu_3_N/Cu(110) surfaces [20]. We note that the location of these dark segments is here linked to the surface orientation by the molecular geometry, where the expected match with the experimental results is fulfilled.

The good match between the key features in experiments and calculations is remarkable as measurements were not performed with a specifically prepared tip. The accidental functionalisation of the tip might have occurred through the adsorption of a FDCA molecule to the tip with the carbonyl moiety facing the surface. This would be in line with earlier NC-AFM experiments of naphthalene tetracarboxylic diimide (NTCDI) adsorbed on Ag-terminated silicon surfaces [37]. In the latter case, the observation of submolecular contrast similar to images acquired with CO-terminated tips has been explained by a tip-adsorbed NTCDI molecule with a CO-like carboxylic oxygen pointing towards the sample [37]. In the present experiments, a similar mechanism with one of the FDCA carbonyl groups pointing towards the sample is plausible. However, the agreement between experiment and simulation might also reflect a more general feature of repulsive mode imaging. While the tip–surface interaction force curves in the attractive region differ significantly from each other for different tip-terminating species, the force curves in the repulsive region are so steep that differences in their details have only minor influence on the NC-AFM contrast. Hence, the NC-AFM contrast observed for imaging with different tip terminations differs only by the distance of the onset of certain features as found for the simulations with CO and xenon tips. In either case, the interaction with the top hydrogen atoms is found to be localised and repulsive and serves as a marker for the hydrogen atom position.

**Figure 3:**
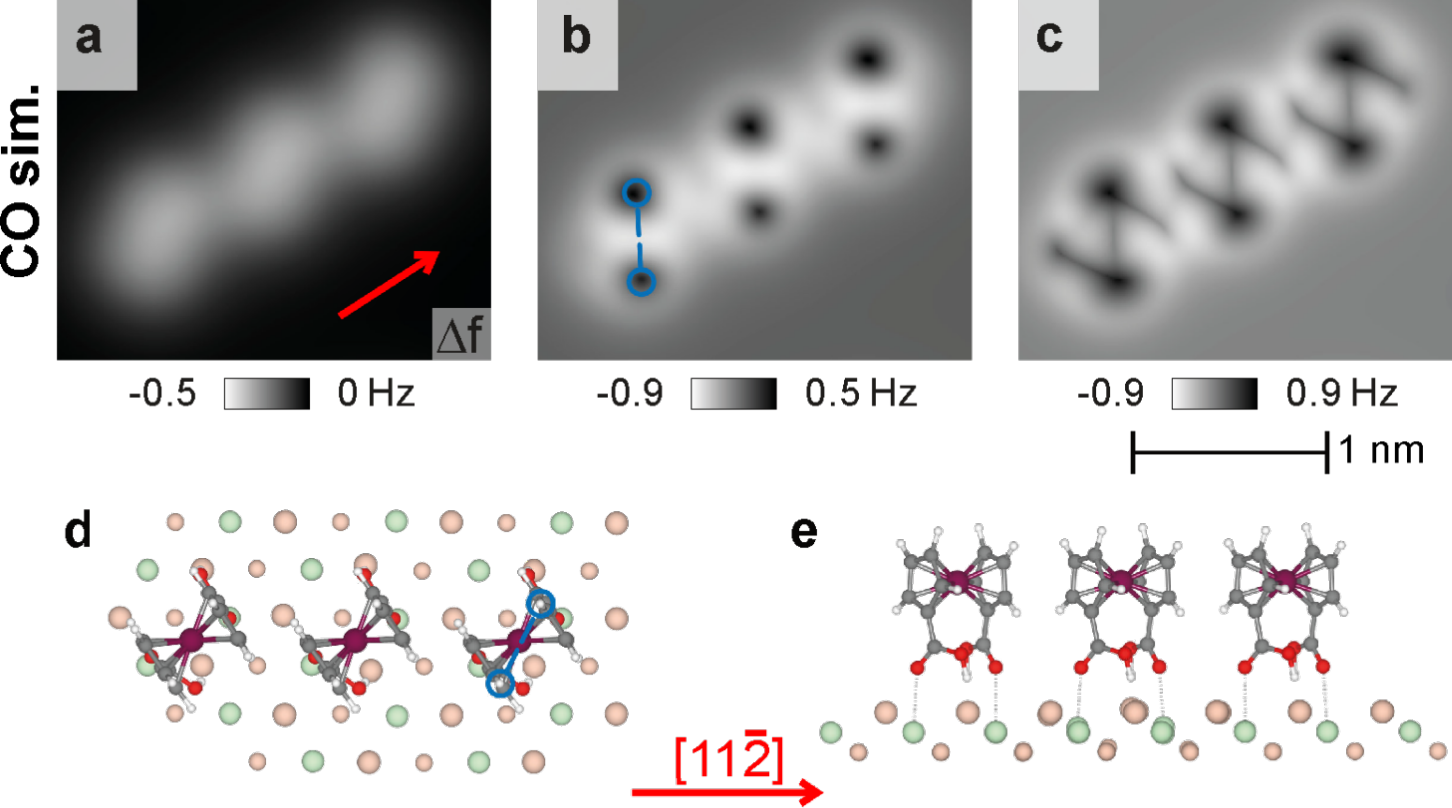
NC-AFM image calculation for a 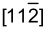 row of FDCA molecules in geo 2. (a–c) Constant-height frequency-shift Δ*f* data (inverted colour scale used) simulated at decreasing tip–sample distances for a row of FDCA molecules, each in geo 2. The red arrows mark the 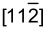 direction and the dashed blue line (forming an angle of 60° with the 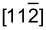 direction) the molecular orientation for geo 2. (d) Top view and (e) side view of the simulation cell using geo 2 from [22].

A previous analysis of the molecular orientations highlighted the presence of a minority population of FDCA molecules exhibiting the adsorption geometry geo 2 with a partly staggered molecular conformation [22]. The protruding hydrogen atoms align in this geometry along a different direction with respect to geo 1 due to the partly staggered conformation of the Cp rings. The corresponding PPM image calculations with identical simulation parameters as before for a CO tip are shown in Figure 3. Although the molecular row still proceeds along the 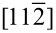 direction, the orientation of the dumbbell shape differs. The angle between the dumbbell-connecting line and the 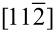 direction reduces from 90° (geo 1 in Figure 2) to 60° (geo 2 in Figure 3). This is a direct consequence of the partly staggered conformation, which moves the top hydrogen atoms to these positions. At further reduced distances (see Figure 3c), lines both along the position of the top Cp bonds and along the iron centre of the FDCA molecule are again revealed.

Experimental examples for the different orientations are given in Figure 1c and Figure 1d. Due to the three-fold rotational symmetry of the CaF_2_(111) surface, a total of six orientations, three for each geometry, are possible and included as sketches in the lower left corners with solid green and dashed blue lines marking the orientation of geo 1 and geo 2, respectively. Molecules of both geometries oriented along one of the possible angles are present in the experimental images, two examples for each geometry are marked. The experimental contrast is similar to the pattern of a single molecule within the calculated images in Figure 2h and Figure 2m for geo 1 and Figure 3b for geo 2.

## Conclusion

We have investigated the origin of the dumbbell shape, which is observed when imaging FDCA molecules on CaF_2_(111) surfaces with NC-AFM. Based on a comparison of experimental constant-height frequency-shift data with image calculations using the probe particle model, we identify repulsive interactions between the tip and the topmost hydrogen atoms of the Cp rings of single FDCA molecules as the fundamental cause for the dumbbell shape. Due to the strong molecule–surface bond, molecules are usually not manipulated while scanning in this repulsive mode. We found tips prepared by standard procedures to be mostly stable and sharp enough to locally enter the repulsive regime. Following the vision of [9], the measurement of the two hydrogen atom positions with respect to the orientation of the CaF_2_(111) lattice allows us to identify the ferrocene conformer and the molecular orientation on the surface. The experimental observation of the dumbbell shape suggests that the prerequisite of a stable tip during repulsive interactions with the hydrogen atoms can easily be fulfilled, even at room temperature. This is in line with previous findings of stable tips for sub-molecular resolution imaging at 77 K [37–38] and 300 K [39]. Due to the predominant repulsive interactions with the chemically rather inert hydrogen atoms, we speculate that the chemical identity of the tip is therefore of secondary importance. Still, the tip has to be sharp enough to allow for resolving the dumbbell shape.

This is a demonstration for identifying hydrogen atoms in a 3D molecule with nonfunctionalised tips as well as the use of hydrogen atoms as markers for the molecular geometry. As the method introduced here is based on an universal imaging mechanism and widely applicable experimental procedures, we expect that the introduced method will allow for the identification of other conformers and, in particular, give further insights into adsorption geometries of complex 3D molecules.

The work further underlines the general applicability of repulsive-mode imaging of molecules independent of a specific tip termination, such as CO, which is hitherto the standard tip for this imaging mode. We propose that high-resolution repulsive-mode imaging of hydrogen atoms is possible with a variety of tips if the following three preconditions are fulfilled: (1) The molecule is sufficiently strongly bonded to the substrate to withstand manipulation due to lateral forces exerted by the approaching tip. (2) The tip atomic arrangement is characterised by a deep minimum of total energy preventing a rearrangement of tip atoms or disintegration if the tip is strained by the interaction with the molecule. (3) The tip end is atomically sharp. The third condition implies the stabilisation of the tip-terminating atom by one or very few bonds directed towards the tip apex. Such a bond will always exhibit sufficient lateral elasticity to yield a sharp contrast feature when the tip terminating atom passes a molecular protrusion.
